# Using Q-methodology to explore people’s health seeking behavior and perception of the quality of primary care services

**DOI:** 10.1186/1471-2458-14-2

**Published:** 2014-01-06

**Authors:** Nazar P Shabila, Namir G Al-Tawil, Tariq S Al-Hadithi, Egbert Sondorp

**Affiliations:** 1 Department of Community Medicine, College of Medicine, Hawler Medical University, Erbil, Iraq; 2 London School of Hygiene and Tropical Medicine, London, UK

**Keywords:** Primary care, Health seeking, Perception, Factors

## Abstract

**Background:**

Information on health seeking behavior and beneficiaries’ perception of the quality of primary care can help policy makers to set strategies to improve health system. With scarcity of research on this particular field in Iraqi Kurdistan region, we sought to explore the patterns of health seeking behavior and perception of the quality of primary care services of a sample of population.

**Methods:**

This explorative study was carried out in Erbil governorate, Iraq. Data were collected using the novel approach of Q-methodology for eliciting subjective viewpoints and identifying shared patterns among individuals. Forty persons representing different demographic and socioeconomic groups and living in different areas of Erbil governorate sorted 50 statements reflecting different aspects of health-seeking behavior and primary care services into a distribution on a scale of nine from “disagree most” to “agree most”. By-person factor analysis through centroid factor extraction and varimax rotation of factors were used to derive latent viewpoints.

**Results:**

Four distinct patterns of health seeking behavior and viewpoints toward the primary care services were identified. People in factor 1 are extremely critical of the services at primary health care centers and are regular users of the private health sector. People in factor 2 positively recognize the services at primary health care centers but mainly turn to inappropriate health seeking behavior. People in factor 3 have satisfaction with the services at primary health care centers with minimal use of these services, but mainly turn to the private sector. People in factor 4 are slightly satisfied with the services at primary health care centers but mainly rely on these services.

**Conclusions:**

This study highlighted the typical characterizations that were associated with each uncovered factor. Informing on the beneficiaries’ concerns about the primary care services can help to improve the system through further exploring the issues raised by the respondents and directing particular action on these issues. The characterizing and distinguishing statements can be used as a set of questions to conduct community-based survey on this important aspect of health services.

## Background

Primary care constitutes an entry point into health services where a wide range of curative and preventive health care services are delivered. Primary care coordinates and integrates the care and is considered the locus of continuing care for most of the health problems that occur in the population [1,2].

The primary care services in Iraq are provided by a network of public primary health care centers (PHCCs) [3]. The private health sector provides mainly curative health services through private clinics and pharmacies. Many nurses and medical assistants’ private clinics are also widely distributed in the governorate that directly prescribe and sell most kinds of medicines [4,5]. The Iraqi health care system particularly its primary care component, was seriously affected by wars, internal conflicts, economic sanctions and political instability over the last few decades [6,7].

Patient’s perspective is increasingly viewed as a meaningful and important indicator of health service quality that should be taken into account as part of a comprehensive assessment of quality of health care [8]. Obtaining beneficiaries’ perception about the quality of health services constitutes an increasingly important outcome in judging the quality of healthcare provision [9].

Utilization of services and perceived quality of health care go hand in hand as beneficiaries’ satisfaction and perceived quality have influence on utilization of services. However, health seeking behavior and utilization of a health care system, public or private, formal or non-formal, are not only related to availability and quality of services, but it is a result of interaction and balance between health needs, health resources, socio-economic and cultural factors [10,11]. Information on health seeking behavior and beneficiaries’ perception about primary care services can help the policy makers set strategies to improve the health system. Due to limited research evidence in this particular field in Iraq particularly in Kurdistan region, we sought to explore the patterns of health seeking behavior and perception of the quality of primary care services of a sample of Iraqi population using the novel approach of Q-methodology.

## Methods

### Setting and time

This study was carried out on a sample of population of Erbil governorate in Iraq from November 4^th^, 2011 through January 10^th^, 2012.

### Q-methodology

Q-methodology provides a foundation for the systematic study of subjectivity [12,13]. Uniquely, Q-methodology combines the strengths of both qualitative and quantitative methods. Typically, in a Q-methodological study people are presented with a sample of statements about some topic, called the Q-set. Respondents, called the P-set, are asked to rank-order the statements from their individual point of view, according to some preference, judgment or feeling about them, mostly using a quasi-normal distribution. By Q-sorting, people give their subjective meaning to the statements, and by doing so reveal their subjective viewpoint. These individual rankings or viewpoints are then subjected to factor analysis [12,14,15].

### Participants

Q-methodology is a kind of exploratory factor analysis, not designed for hypothesis testing. Therefore, it is not subjected to sample size estimation. The number of participants is usually, but unnecessarily, smaller than the Q-set [16]. The aim is to have four or five persons defining each anticipated viewpoint, which are often two to four, and rarely more than six. Accordingly, breadth and diversity of viewpoints is probably best achieved when a participant group contains between 40 and 60 participants [17]. Therefore a sample size of 40 persons was selected.

Samples in studies employing Q-methodology need to be carefully selected rather than randomized so that variability in a specific case or situation can be analyzed [18]. Therefore, a purposive sample of individuals who were potentially representative of the various issues of the study topic and those who could provide the best insights on the study topic was recruited. The aim was to recruit individuals of both sexes and representing different age groups, employment statuses and professions, socioeconomic levels and geographical areas of Erbil governorate so that most patterns of health seeking behavior and perception of the quality of primary care services could be identified. The employed individuals were selected from four governmental institutions, each from a different part of Erbil city. At each institution, six to eight employees from both sexes and from different positions and professions were invited to participate in the study. Another purposive sample of six unemployed/casual labors from different areas of Erbil city was selected. Finally, six individuals, three employed and three unemployed/casual labors, from semi-urban and rural areas away from Erbil city were recruited.

### Identification of statements

A pool of statements that could potentially describe and sufficiently represent the topic of investigation was generated. To determine the issues and viewpoints concerning the health seeking behavior and the quality of primary care services, in-depth interviews were conducted with four purposively selected persons representing different professions, socioeconomic levels and geographical areas of Erbil governorate. Moreover, notes were taken from individual discussions and general talks with patients visiting PHCCs, physicians’ private clinics, a nurse private clinic and a private hospital. Statements made by participants about their health seeking behavior and their perception of the quality of primary care services in different types of health facilities were recorded.

### Compiling the Q-sample statements

As a result of the statement identification step, 132 statements were extracted representing the views of 42 respondents. All statements were reviewed for similarities and differences. Responses that were repeated were discarded. Two members of the research team made independent decisions about repetitive responses. Their results were compared, and the two researchers discussed any responses where there was lack of agreement until consensus was reached. The expressions of the respondents were used; only grammar of the statement was edited. Finally, 50 statements were selected.

### Creating the Q-sort

Once the 50 statements were selected, they were numbered randomly and typed onto small cards with one statement per card. The statements were originally made in Kurdish language. After the Q-sample was created, the Q-sort was developed, which involved creating a quasi-normal distribution with 50 cells equal to the number of the Q-sample statements. This constituted the data collection instrument for the study. The Q-sort was pilot-tested on a convenience sample of four persons. Feedback was collected on the clarity of statements, ease of the task, length of time for completion and general suggestions about the process. Modifications were made to the instrument and the instructions accordingly.

### Data collection

The purpose of the study and instructions for completing the task were explained to each participant before obtaining his consent. Each participant was asked to sort the cards into nine piles, from -4 (disagree most) to +4 (agree most), in relation to his/her perception about different aspects of health seeking behavior and perception toward the quality of primary care services and according to the Q-sort table. Clear step by step instructions were provided to each participant about how to sort the cards before leaving him/her to complete the sorting alone. The Research Ethics Committee of Hawler Medical University approved this study.

### Data analysis

The PQMethod 2.11 program was used for the analysis of Q-sorts [19]. The prominent common viewpoints, known as factors, were extracted using centroid factor extraction and varimax rotation. Stringent criteria were used for factor selection with factors representing at least two defining sorts and having eigenvalues greater than one were extracted [20]. Defining sorts are Q-sorts that are both significant for the factor but not significant on any other factor. Those Q-sorts that achieved absolute factor loading of 0.365 or above on a given factor, which suggests high significance (p < 0.01), were included into that particular factor [17]. An explanation of how this is calculated is shown Additional file 1. The Q-sorts that had significant loading on more than one factor were not classified into any factor. An eigenvalues is the sum of squared loadings for a factor; it conceptually represents the amount of variance accounted for by a factor [21]. However, several different factor solutions were examined for obtaining the most meaningful, consistent and coherent factors. The resultant factors represent sorts made by individuals who have responded in essentially the same way. When all of the weighted average scores of the statements of each factor were obtained from the correlation matrix, the statements were arranged in order of descending scores. This arrangement formed the composite statement array for that factor. To facilitate comparisons between factors, composite statement scores were transformed back into the whole-number scores (+4, +3, +2, etc.) used in the original sorting process. Factor arrays provide a conceptual representation of the factor [22].

Each factor or viewpoint was interpreted subjectively by examining its characterizing and distinguishing statements. The characterizing statements of a factor are those with a rank value of '+4’, '+3’, '-3’, '-4’ in the factor arrays. A distinguishing statement for a factor is a statement whose score on that factor is significantly different from its score on any other factor. Distinguishing statements that are significant at p < 0.05 are highlighted with asterisk (*), and those at p < 0.01 are highlighted with double asterisk (**) in the results section. Finally a conceptual interpretation was developed to capture the essence of the viewpoints being endorsed.

## Results

Forty persons participated in the study. Their ages ranged between 19 and 62 years with a mean ± SD of 37.0 ± 9.2 years. Details of sociodemographic characteristics of the participants are shown in Table 1.

**Table 1:** Socio-demographic characteristics of the participants

**Characteristics**	**No.**	**(%)**
**Gender**		
Male	20	(50.0)
Female	20	(50.0)
**Age group (year)**		
<30	9	(22.5)
30-39	14	(35.0)
40-49	14	(35.0)
≥50	3	(7.5)
**Marital status**		
Single	8	(20.0)
Married	32	(80.0)
**Education**		
Primary school	5	(12.5)
Secondary school	14	(35.0)
Higher education	17	(42.5)
Postgraduate study	4	(10.0)
**Family size**		
1-3	13	(32.5)
4-5	14	(35.0)
≥6	13	(32.5)
**Number of children in the family**		
0	6	(15.0)
1	7	(17.5)
2	9	(22.5)
3	7	(17.5)
≥4	11	(27.5)

Analysis of the participants’ Q-sorts resulted in a four factor solution, i.e. four distinct patterns of health seeking behavior and perception of the quality of the public primary care services (Table 2). These four viewpoints included; (i) extremely critical, private sector users, (ii) highly positive, inappropriate health seeking, (iii) some satisfaction and minimal users and (iv) slight satisfaction and regular users.

**Table 2:** Q-set statements and factor array

**#**	**Statement**	**Factor**			
		**1**	**2**	**3**	**4**
1	Women adequately use antenatal care services at PHCCs	0**	3	2	3
2	I only visit PHCCs for vaccination of children	-1	-1	-4	-3
**3**	**It is not easy to call a family doctor or a doctor we know to get advice for any illness**	**2**	**1**	**3**	**2**
4	The working hours of PHCCs are suitable to most people	-1	2	-1	0
5	Necessary laboratory investigations are not available at PHCCs	1	1	-3**	1
6	There is good organization of service provision at PHCCs	-2**	2	0*	1
7	People have easy access to health services at PHCCs	0**	3	3	1*
8	Doctors focus too much on medication and neglect the psychosocial aspects of patients	2	0	-3	-2
9	Physicians in PHCCs excessively refer patients to specialists for their health problems	1	1	-1*	-3**
10	For simple illness like flu I visit a private nurse clinic	1	4**	0*	0
11	There are convenient waiting amenities at PHCCs	-2**	4**	1	0
12	Having the PHCCs run mainly by general practitioners and not specialists is an important reason for not using these services.	3**	-1	1**	-1
13	PHCCs are mainly used by poor people	3	0*	1*	3
14	Sometimes I make some general investigations before or even without consulting a doctor	1	-3	1	-3
15	If I think the disease is serious I will consult a private physician	4	-3**	4**	4
16	When I am really ill, I visit a PHCC to get consultation	-1	-1	-3**	1*
17	I seek family member advice on decision making for source of health care	-1	-2	1**	-1
18	If I feel sick I try to consult a specialist rather than a general practitioner	3	-2**	3	1
19	People consider the doctor that prescribe more drugs particularly injections a better doctor	0**	-3	-1	-2
20	Doctors at PHCCs instead of dispensing available drugs prescribe costly medicines from the private sector	1*	-4**	-2*	0*
21	Doctors in general provide sufficient explanation of the disease and treatment	-1*	-4*	1*	0*
22	Health care providers at PHCCs are not competent and are not trained well	1	-2	0	-1
23	Health care professionals at PHCCs are adequately understanding and empathetic towards people	-2	0	-1	0
24	I sometimes visit a hospital without getting a referral from PHCC	0	0	2*	0
25	I do not visit PHCCs at all	0	-2	-3	-4**
26	I usually visit PHCC to get referral to a hospital	0	-3**	0	1**
**27**	**We commonly visit a private pharmacy for consultation and getting treatment**	**-1**	**-2**	**-2**	**-1**
28	In PHCCs, there is enough opportunity for patients to ask questions or obtain explanations for their health problems	-3*	0	-2*	1
29	The hygienic situation at PHCCs is well maintained	-3	-2	1	2**
30	Patient’s privacy and confidentiality are not adequately respected in PHCCs	1	-1	-1	0
31	When I feel sick I take left-over drugs by myself	-2*	2*	-1	-4**
32	I use private nurse/medical assistant clinic for being near and less costly	0	3*	-4**	2
33	PHCCs have only analgesics and no real treatment	2	0	0	4*
34	For some types of illness I use traditional healers	-4	2**	-1*	-3
35	Physicians in private sector understand the financial condition of patient and prescribe treatment accordingly	-2	-1	0	-1
36	Physicians at PHCCs provide enough care and time to patients	-3**	2	0*	2
37	When I have any health problem, initially I either do not do anything or try some home remedies	1	1	1	3
38	At private clinic physicians spend more time and provide better care to patients than in PHCCs	4*	-1	2*	1
39	Health education service is provided adequately in PHCCs	-3	2**	-2	-1
40	Health expenditures cause financial burden on the family	2	3	4	-2**
41	People prefer private physicians clinics due to lack of drugs in PHCCs	2	0*	2	2
42	For any illness, we make an early consultation to avoid taking risk	0	1	2	2
43	The families who can afford the expenditure prefer private health facilities and private doctors for seeking treatment over public facilities	3	0	2	0
44	For children illness we visit a PHCC for consultation	-1**	1	-2**	3*
45	Physicians at PHCCs listen to the problems patiently before prescribing treatment	-4**	1	0	-2*
46	We consult a private specialist of our choice according to the nature of the disease we have	2	-1	3	0
47	I ask physician or health staff to prescribe or administer injections	0	0	-2	-1
48	For some types of illness we turn to shrines or local religious methods for relief	-1	0*	-1	-2
49	The waiting time for having services at PHCCs is appropriate	-2	1	0	-1
50	We very rarely seek after-hour services or immediate medical advice like visiting emergency hospital	0	-1	0	-2

* Distinguishing statement significant at <0.05; ** Distinguishing statement significant at <0.01; Bold type indicates consensus statement.

The four factors were defined by 28 participants (70.0%), whereas seven participants did not load significantly on any of the factors and five participants were confounded. The viewpoints had between two and 14 defining variables (i.e. responses significantly associated with the viewpoint). Together, they account for 36% of the variance in the Q-sorts. The socio-demographic characteristics and factor loading for each participant on each of the four factors are shown in Additional file 1.

### Factor 1: extremely critical, private sector users

Fourteen participants loaded significantly onto factor 1; eight males and 6 females, two singles and 12 married, three at managerial positions, six regular employees and five unemployed/casual labors, and seven having higher education. These participants strongly emphasized the negative aspects of the services at the PHCCs and preferred seeking healthcare in physicians’ private clinics.

Participants loading on this factor were in favor of consulting a private physician for serious diseases (statement (S)15: (rank) +4) and consulting a specialist rather than a general practitioner (S18: +3) as they thought that physicians at private clinics spend more time and provide better care to patients than those in PHCCs (S38: +4)**. They thought that the families who can afford expenditures prefer private health facilities and private doctors for seeking treatment over public facilities (S43: +3). They were not supportive of using inappropriate health services like taking left-over drugs by themselves (S31: -2)** or turning to traditional healers for some types of illnesses (S34: -4). They claimed that they do not visit PHCCs for children illness (S44: -1)** and considered PHCCs mainly used by poor people (S13: +3). They attributed poor use of PHCC services to that PHCCs were run mainly by general practitioners and not specialists (S12: +3)**. They also thought that women do not adequately use antenatal care services at PHCCs (S1: 0)**.

Compared to other groups, this group of participants strongly emphasized the negative aspects of the services provided at PHCCs. They were particularly concerned about the poor patient-provider interaction. This is illustrated in the following ranking of items:

'Physicians at PHCCs listen to the problems patiently before prescribing treatment’ (S45: -4)**

'Physicians at PHCCs provide enough care and time to patients’ (S36: -3)**

'There are enough opportunities for patients at PHCCs to ask questions or obtain explanations for their health problems (S28: -3)*

'Doctors at PHCCs instead of dispensing available drugs prescribe costly medicines from the private sector’ (S20: 1)*

'Health education service is provided adequately at PHCCs’ (S39: -3)

These participants also underlined the poor access to services and poor organization of service provision at PHCCs:

'There is good organization of service provision at PHCCs’ (S6: -2)**

'There are convenient waiting amenities at PHCCs’ (S11: -2)**

'People have easy access to health services at PHCCs’ (S7: 0)**

'The hygienic situation of PHCCs is well maintained’ (S29: -3)

These participants considered doctors insufficiently explain the disease and the treatment (S21: -1)*. However, they generally defined the doctor is good as long as he prescribe injections for them (S19: 0)**.

### Factor 2: highly positive, inappropriate health seeking

Two participants loaded significantly onto factor 2; one single and one married, one at managerial position and one regular employee, and both having higher education. This group of participants strongly emphasized the positive aspects of the services at PHCCs but they generally turn to inappropriate health seeking behavior.

Participants loading on this factor were in favor of frequently turning to inappropriate health seeking behavior like utilizing private nurse clinics, traditional healer or taking left-over drugs:

'For simple illness like flu I visit a private nurse clinic’ (S10: +4)**

'I use private nurse/medical assistant clinic for being near and less costly’ (S32: +3)*

'For some types of illness I use traditional healers’ (S34: +2)**

'When I feel sick I take left-over drugs by myself’ (S31: 2)*

'For some types of illness we turn to shrines or local religious methods for relief’ (48: 0)*

They were not much concerned about consulting a specialist rather than a general practitioner when feeling ill (S18: -2)** or consulting a private physician if they think the disease is serious (S15: -3)**. Compared to other groups, participants loading on this factor did not think that PHCCs are mainly used by poor people (S13: 0)*. These participants did not seem to visit PHCCs to get referral to a hospital (S26: -3)** or make some general investigations before or even without consulting a doctor (S14: -3).

Even though these participants did not seem to regularly use the services at the PHCCs, they underlined many positive aspects of these services:

'People have easy access to health services at PHCCs’ (S7: +3)

'Women adequately use antenatal care services at PHCCs’ (S1: +3)

'Health education service is provided adequately in PHCCs’ (S39: +2)**

'There are convenient waiting amenities at PHCCs’ (S11: +4)**

'Doctors at PHCCs instead of dispensing available drugs, prescribe costly medicines from the private sector’ (S20: -4)**

'There is good organization of service provision at PHCCs’ (S6: +2)

'People prefer attending private physicians’ clinics due to lack of drugs at PHCCs’ (S41: 0)*

These participants thought that doctors do not sufficiently explain to patients about the disease and the treatment (S21: -4)**. They did not consider doctors who prescribe more drugs, particularly injections, better doctors (S19: -3). They were concerned about health expenditures that cause financial burden on the family (S40: +3).

### F3: some satisfaction and minimal users

Seven participants loaded significantly onto factor 3; three males and four females, two singles and five married, four at managerial positions and three regular employees, and all having good education. This group of participants showed some satisfaction with the services at PHCCs, but they minimally use these services and mainly prefer attending private sector.

Participants loading on this factor were in favor of consulting private physicians for serious diseases (S15: +4)**, consulting a specialist rather than a general practitioner (S18: +3) and consult a private specialist of their choice according to the nature of the disease they have (S46: +3). They thought that at private clinics physicians spend more time and provide better care to patients than in PHCCs (S38: +2)*. Having the PHCCs run mainly by general practitioners and not specialists was an important reason for not using these services by these participants (S12: +1)**. They also thought that there is enough opportunity for patients at PHCCs to ask questions or obtain explanations for their health problems (S28: -2)*.

These participants seemed to seek family member advice on decision making for source of health care (S17: +1)**. They claimed that they do not visit PHCCs to get consultation when they are really ill (S16: -3)** or for consultation for children illness (S44: -2)**, however they seemed to visit PHCCs sometimes as they disagreed with the statement of not visiting PHCCs at all (S25: -3) and only visiting PHCCs for vaccination of children (S2: -4).

Compared to other groups, these participants were less concerned about using traditional healers for some types of illness (S34: -1)* or visiting private nurse clinics for simple illness like flu (S10: 0)*. Indeed, they strongly disagreed with using private nurse/medical assistant clinic for being near and less costly (S32: -4)**. They also revealed some agreement with the statement that PHCCs are mainly used by poor people (S13: +1)* and seemed to visit hospitals even without getting referral from PHCCs (S24: 2)*.

These participants emphasized the easy accessibility of services at PHCCs and showed some satisfaction with organization of these services:

'People have easy access to health services at PHCCs’ (S7: +3)

There is good organization of service provision at PHCCs’ (S6: 0)*

'Physicians in PHCCs excessively refer patients to specialists for their health problems’ (S9: -1)*

'Doctors at PHCCs prescribe costly medicines from the private sector instead of dispensing available drugs’ (S20: -2)*

'Necessary laboratory investigations are not available at PHCCs’ (S5: -3)**

'Physicians at PHCCs provide enough care and time to patients’ (S36: 0)*.

These participants thought that doctors in general provide sufficient explanation of the disease and treatment (S21: +1)* and they do not focus too much on medication, but on the psychosocial aspects of patients (S8: -3). They pointed out to the fact that health expenditure is a financial burden on the family (S40: +4).

### F4: slight satisfaction and regular users

Five participants loaded significantly onto factor 4; two males and three females, two singles and three married, three regular employees, one at managerial position and one unemployed/casual labor. These participants were partially satisfied with the services at PHCCs and were usual users of these services.

Participants loading on this factor claimed that they initially either do not do anything or try some home remedies when having a health problem (S37: +3). While these participants thought that PHCCs are mainly used by poor people (S13: +3), they seemed to seek healthcare at PHCCs more often in comparison with the other groups:

'For children illness we visit a PHCC for consultation’ (S44: +3)*

'When I am really ill, I visit a PHCC for consultation’ (S16: +1)*

'I do not visit PHCCs at all’ (S25: -4)**

'I only visit PHCCs for vaccination of children’ (S2: -3).

'Women adequately use antenatal care services at PHCCs’ (S1: +3)

However, they pointed out to visiting PHCCs sometimes for referral to a hospital (S26: +1)**. They preferred consulting a private physician for serious diseases (S15: +4).

These participants typically were not in favor of taking left-over drugs by themselves when feeling ill (S31: -4)** or turning to traditional healers for some types of illness (S34: -3). They also did not seem to make some general investigations before or even without consulting a doctor (S14: -3).

This group of participants emphasized some positive aspect of the services at the PHCCs:

'People have easy access to health services at PHCCs’ (S7: +1)*

'Physicians in PHCCs do not excessively refer patients to specialists for their health problems’ (S9: -3)**

'The hygienic situation of PHCCs is well maintained’ (S29: +2)**

They also highlighted some impediments for using the services at PHCCs:

'PHCCs have only analgesics and no real treatment’ (S33: +4)*

“Doctors at PHCCs instead of dispensing available drugs prescribe costly medicines from the private sector’ (S20: 0)*

'Physicians at PHCCs listen to the problems patiently before prescribing treatment’ (S45: -2)*

These participants were somewhat satisfied with doctors for sufficient explanation of the disease and treatment (S21: 0)*. They did not think that health expenditure is a financial burden to the family (S40: -2)**.

## Discussion

This study identified four distinct patterns of study participants’ health seeking behavior and perception toward the quality of primary care services. People in factor 1 reflected a typical model of health seeking behavior that result from clear dissatisfaction with the available public services that eventually lead to the use of private services [23]. The private health services in Iraq are widely used particularly by affluent people since these services are widely available and are easily accessible [24]. The main benefits sought from the private sector by this group of respondents were consulting specialists because they are not always available in PHCCs and obtaining good quality of care and patient-provider interaction. Previous studies from Iraq have reported a generally high preference to using private health services for primary care (53-60%) compared with PHCCs (16-21%) [24,25].

Preference of consulting a specialist rather than a general practitioner was common for people in factors 1, 3 and 4. There are a number of factors that result in this preference. The Iraqi health system has been historically based on a hospital-oriented, capital-intensive model that was increasingly depending on specialist doctors. Unlike many western countries, general practitioners in Iraq lack formal postgraduate training. Recently, there is increasingly unusual assignment of specialists to PHCCs due to their increasing numbers and limited capacity of the available hospitals to accommodate them [26]. With the current health system in Iraq, patients can visit any specialist in the private sector without a need for referral. All these factors have made Iraqi people increasingly dependent on specialist doctors.

The critics to the services at PHCCs particularly by the participants loading on factor 1 were mainly related to poor patient-provider interaction and poor access to and organization of services. Other studies from Iraq have shown a good overall satisfaction with PHC services, but reported also poor patient-provider interaction and poor organization of services particularly the long waiting time [24,25]. Whereas availability and physical access is important, patients’ perspectives on the quality of care as experienced through the patient-provider encounter play a major role in health seeking behavior [27].

It was interesting to observe how people in factor 2 in spite of having a high satisfaction with the services at PHCCs, turn mainly to inappropriate health seeking behavior. There might be other barriers to use the services at PHCC that might not be included in the list of statements used for this Q-study or these people might have other believes that could not be detected by this study. It would be important to explore the reasons behind poor use of PHCC services by this group of people. The different models of health seeking behavior have suggested many other factors other than satisfaction with services that can influence the health seeking behavior of the individual and the community [23,28].

People in factor 3 showed some similarities to those in factor 1 in terms of using mainly private services. However, these people have some satisfaction with the services at PHCCs and also sometimes use these services in addition to turning to inappropriate health seeking behavior. It is striking to observe such diverse health seeking behavior among this group of participants. This needs to be deeply explored to identify the main factors and determinants associated with each health seeking behavior adopted by this group of people.

People in factor 4 although have only slight satisfaction with the primary care services, they mainly use these services. This could be the kind of viewpoint of many poor people who cannot afford the services in the private sector particularly that this group thought that PHCCs are mainly used by poor people. However, we cannot reach such general conclusion from this study as the sample in Q-studies cannot be generalized. This group believed that there was no financial burden on families as long as they use health services of PHCCs that are nearly free of charge. All other factors strongly emphasized the financial burden resulting from health expenditures attributed to increasingly depending on private services. Difficulty in affording health care was also reported by another study from Iraq [24].

Studies employing Q-methodology can be helpful in exploring the different patterns of health seeking behavior of population and their relation with perceptions of the quality of the available services. Mixed quantitative and qualitative approaches have been recommended to help in capturing both prevalence of behavior according to specific health conditions and the rationale for specific health seeking behavior pathways [28]. Q-methodology can be of added value in this respect as it combines the strengths of both qualitative and quantitative research [29]. Q-studies can also direct future quantitative and qualitative research in this field. Presentation of the uncovered four viewpoints on the basis of the characterizing and distinguishing statements to a representative sample of population could determine different socio-demographic factors associated with each viewpoint. In-depth qualitative studies can explore different social and cultural factors responsible to the uncovered viewpoints.

### Limitations

This research has some potential limitations. It is mainly an exploration of the patterns of health seeking behavior and the range of viewpoints about the primary care services that are embedded in the population. The study is not meant to be representative as Q-studies are explorative rather than potential generalizable studies. It is certainly difficult to argue at this stage that this is the definitive range and variety of patterns and viewpoints among the population on the basis of one explorative study. To confirm the validity of these patterns and viewpoints, replication of the study is needed particularly in other governorates and other parts of Iraq. While this study tried to explore the association between the patterns of health seeking behavior and the perception of the quality of primary care, it should be noted that perception of quality of services alone cannot determine the health seeking behavior of an individual or a community but there are many other factors that can influence the health seeking behavior [23,28]. Another limitation of the use of Q-methodology is acquiescence bias (also known as agreeing-response bias) which is the propensity for participants to agree with a statement or assertion presented to them regardless of its contents. This type of bias is believed to be an important limitation of this type of agree-disagree and other Likert-scale format questions [30]. For these types of agree-disagree questions a midpoint can be seen as an easy option to take when a respondent is unsure, so it is questionable whether it is a true neutral option. People may be less discriminating and not take the time to weigh the merit of each response category [31]. Lastly, the viewpoints of the poorer and less educated people might have been overlooked as administration of Q-sort requires the respondent to have a certain level of education.

## Conclusions

A range of four diverse patterns of health seeking behavior and perceptions of the quality of primary care services were uncovered by using Q-methodology. The study highlighted the typical characterizations that are associated with each viewpoint and helped in identifying the main issues of concern to this important group of stakeholders in terms of problems facing these services and the obstacles for their utilization. Informing on the beneficiaries’ perceptions of the primary care services can help to improve the system through further exploring the issues raised by the respondents and directing particular action on these issues. These findings can contribute to a better understanding among health policy makers and primary care managers of populations’ concerns about the primary care services and might assist in influencing change management. These findings can direct more comprehensive quantitative and qualitative research to better understand the factors associated with each viewpoint. The characterizing and distinguishing statements can be used as a set of questions to conduct community-based survey to assess the health seeking behavior of the population and to identify the socio-demographic factors that are associated with each pattern of health seeking behavior and perception of primary care services.

## Abbreviations

PHCC: Primary health care center.

## Competing interests

The authors declare that they have no competing interests.

## Authors’ contributions

NPS, NGAT and TSAH conceptualized the study. NPS, NGAT and ES designed the study. NPS and NGAT collected the data. NPS and ES carried out data analysis and interpretation. NPS, NGAT and TSAH prepared the manuscript. ES and TSAH extensively reviewed and edited the manuscript. All authors read and approved the final manuscript.

## Pre-publication history

The pre-publication history for this paper can be accessed here:

http://www.biomedcentral.com/1471-2458/14/2/prepub

## Supplementary Material

Additional file 1 Participants’ characteristics and factor loading on the four factors. Click here for file
