# Supplementary material for: Using Q-methodology to explore people’s health seeking behavior and perception of the quality of primary care services
Source: BMC Public Health. 2014 Jan 6;14:2. doi: 10.1186/1471-2458-14-2 (PMC3882479; doi:10.1186/1471-2458-14-2)
Supplement: Additional file 1 — Participants’ characteristics and factor loading on the four factors. [file 1471-2458-14-2-S1.pdf]

Additional file 1 - Participants' characteristics and factor loading on the four factors

Participant No.	Age	Sex	Marital	Job type	Education	Family size	No. of children	Factor			
								1	2	3	4
P1	28	Female	Single	None/Temporary	Secondary	8	6	**0.825**[x]	-0.063	0.225	0.127
P18	42	Male	Married	Managerial	Higher	4	2	**0.809**[x]	-0.111	-0.4	0.007
P2	29	Female	Married	Regular	Higher	3	1	**0.671**[x]	-0.304	0.059	-0.198
P24	32	Male	Married	Regular	Secondary	5	3	**0.659**[x]	0.053	0.163	0.332
P29	37	Female	Married	None/Temporary	Primary	5	3	**0.659**[x]	0.053	0.163	0.332
P19	44	Male	Married	None/Temporary	Secondary	6	4	**0.658**[x]	0.078	-0.186	-0.08
P33	27	Female	Married	Managerial	Higher	4	2	**0.651**[x]	-0.169	0.331	0.029
P21	46	Male	Married	Regular	Secondary	4	2	**0.607**[x]	-0.351	0.33	0.133
P5	56	Male	Married	Regular	Higher	12	10	**0.597**	**0.366**	0.031	0.007
P7	38	Female	Married	Regular	Secondary	3	1	**0.554**[x]	-0.062	0.06	0.178
P31	38	Male	Married	Managerial	Higher	3	1	**0.538**[x]	0.133	0.143	-0.034
P39	41	Male	Married	None/Temporary	Primary	6	4	**0.513**	-0.082	**0.378**	-0.025
P6	31	Female	Single	Regular	Higher	5	3	**0.446**[x]	0.111	0.012	-0.022
P36	30	Female	Married	Regular	Higher	3	1	**0.441**	-0.001	**0.385**	-0.105
P22	41	Male	Married	Regular	Secondary	4	2	**0.427**[x]	-0.209	0.103	-0.103
P13	43	Male	Married	None/Temporary	Secondary	8	6	**0.391**[x]	-0.017	0.342	-0.031
P28	37	Male	Married	None/Temporary	Primary	4	2	**0.388**[x]	0.261	-0.029	-0.067
P17	31	Male	Married	Managerial	Higher	3	1	0.026	**0.525**[x]	-0.033	-0.14
P20	45	Male	Married	None/Temporary	Secondary	6	4	0.165	**0.497**	**0.459**	0.303
P23	43	Female	Single	Regular	Postgraduate	10	3	0.07	**-0.425**[x]	0.062	-0.332
P26	26	Female	Married	Regular	Secondary	2	0	0.234	**-0.37**	**0.463**	-0.104
P16	42	Female	Married	Regular	Secondary	2	0	0.129	0.182	**0.584**[x]	0.017
P37	44	Male	Married	Managerial	Postgraduate	4	2	0.184	-0.001	**0.533**[x]	-0.179
P9	41	Female	Married	Regular	Secondary	3	1	-0.095	0.189	**0.558**[x]	0.201
P32	32	Female	Single	Managerial	Higher	6	4	0.085	-0.008	**0.551**[x]	0.176
P35	34	Male	Married	Managerial	Higher	3	1	-0.102	-0.185	**0.407**[x]	0.14

Participant No.	Age	Sex	Marital	Job type	Education	Family size	No. of children	Factor			
								1	2	3	4
P11	19	Female	Single	Regular	Secondary	1	0	0.338	-0.204	**0.400**[x]	-0.222
P34	45	Male	Married	Managerial	Higher	5	3	0.161	0.018	**0.385**[x]	-0.093
P12	30	Male	Single	Regular	Higher	6	4	-0.04	0.002	0.343	**0.745**[x]
P30	27	Female	Married	Managerial	Higher	4	2	0.169	-0.188	0.26	**0.576**[x]
P4	25	Male	Single	Regular	Higher	6	3	0.017	0.163	-0.144	**0.494**[x]
P15	52	Female	Married	Regular	Secondary	4	2	0.156	0.094	-0.116	**0.441**[x]
P40	34	Female	Married	None/Temporary	Primary	5	3	0.132	0.354	0.241	**0.366**[x]
P25	35	Female	Married	Regular	Secondary	4	2	0.361	0.133	0.296	-0.039
P27	41	Male	Married	Managerial	Higher	6	4	0.179	0.173	0.102	0.029
P10	48	Female	Married	Regular	Postgraduate	2	0	0.093	0.032	0.117	-0.319
P3	23	Male	Married	None/Temporary	Primary	2	0	0.077	-0.004	0.211	0.303
P38	27	Female	Married	Regular	Higher	2	0	-0.015	-0.181	0.125	0.155
P14	62	Male	Married	Regular	Higher	6	5	-0.041	0.2	0.038	0.015
P8	35	Female	Single	Regular	Postgraduate	8	6	-0.071	-0.039	-0.024	0.093
Eigenvalues								7.4	3.1	2.2	1.6
Defining sorts								14	2	7	5
% Explaining variance								16	5	9	6
% Explaining variance cumulative									21	30	36

Bold figures indicate significant loadings. Significance at the 1% level is taken as a factor loading greater than (2.58 x 1√n), where n= the number of statements – so in this case significant loadings are those higher than 0.365.

[x] indicates defining sorts
